# Some Points Worth Considering in the Treatment of Acute Gonorrhea

**Published:** 1908-10

**Authors:** B. F. Senftenberg, J. S. Kreshover

**Affiliations:** Attending Surgeon Beth Israel Dispensary, New York; Assistant Attending Surgeon Beth Israel Dispensary and Bellevue Out-Patient Department, New York


### BUFFALO MEDICAL JOURNAL.

VOL. LXIV. OCTOBER, 1908. No. 3

### ORIGINAL COMMUNICATIONS.

### Some Points Worth Considering in the Treatment of Acute Gonorrhea.

INDEX THROUGH

BY B. F. SENFTENBERG, M.D.
Attending Surgeon Beth Israel Dispensary, New York,
and
J. S. KRESHOVER, M.D.
Assistant Attending Surgeon Beth Israel Dispensary and
Bellevue Out-Patient Department, New York.

GONORRHEA is one of the most virulent and persistent diseases with which mankind is afflicted. It attacks chiefly the mucous membrane of the urethra and is often very rebellious to all known treatment. A urethritis may be either simple or non-gonorrheal, but usually it is gonorrheal in character and is essentially of sexual origin. Although the gonococcus, which is a diplococcus, attacks chiefly the mucous membrane of the urinary tract, it may often be carried through the circulation and involve the various joints and may in some rare cases even attack the endocardium and result in death. The inflammatory process thus produced by the gonococcus, if unchecked by early and appropriate treatment, may ascend in the case of both male and female, and invade the parts that are in immediate or remote anatomical relation. That gonorrhea is one of the most widespread and serious of infectious diseases cannot be denied, and that in its far-reaching effects it does not fall very far short of syphilis in importance, must be admitted by all physicians who have intelligently grasped the situation. Surely not less than 90 per cent. of the male population in the larger cities of the world are said to have or have had gonorrhea, and at least 50 per cent. of the female, and much of the male sterility may be ascribed to the gonococcus.

Since we know that the diplococcus of Neisser is the sole cause of specific urethritis, and that it has a most marked tendency to insinuate itself and proliferate in the folds of the urethra and particularly in the prostatic portion of the urethra, it follows that the destruction of this germ should be the main object of our treatment, and this is to be accomplished with as little injury to the tissues as possible. No case of gonorrhea can be looked upon as cured in which although the discharge has ceased, the gonococcus can be found in the secretion of the urethra after a thorough massage of the prostate. Such examinations should be carefully made even after all apparent discharge has ceased, a great many times over a period of weeks and even months, before a physician is justified in declaring a case cured. To undertake the treatment of a gonorrhea without the aid of a microscope is in the vast majority of cases to invite a failure in the start. The secretions and the urine in the acute stage should be examined from day to day and the progress of the case noted.

Realising the latent powers for evil of the gonococcus we should therefore strive the more by the most thorough, judicious and long continued treatment and observation of this disease, to eradicate it and prevent this germ from taking a firm foothold in any of the folds or crypts of the tissues, there to spring into a renewed virulence under favorable circumstances. Just so long as cases of gonorrhea are improperly and insufficiently treated, will the very common uncured gonorrhea prevail, and not only may the subject's well being and even life be endangered, but he or she may transmit the dreadful pestilence to others. This, then, and the fact that to stop a discharge is not to cure a gonorrhea should be strongly impressed upon the minds of our patients and of the laity in general.

So far as treatment is concerned, to repeat again, it is essential for us to bear in mind that in the great majority of acute cases, the disease is limited to the anterior portion of the urethra, and that if we can bring our patient at once under proper care we can generally hold the disease in check so that the immediate anatomical parts will not be infected. As to proper treatment of this affection there is no unanimity of opinion, each method of treatment has its advocate and likewise its strong opponents. Nearly all, however, are agreed that no active treatment should be undertaken during the acute inflammatory stage; here cleanliness, restricted diet and rest should be enforced. When the sharp inflammatory symptoms have subsided, local treatment is begun and the most favored remedy at our disposal is protargol. The discharge should be examined, two-glass test made, and the character and the nature of the infection thus determined. The bowels should be frequently opened with saline purgatives, and copious urination should be induced for the washing away of as many gonococci as possible by the partaking of large quantities of carborated or plain water. Sexual excitement and violent exercise it is needless to say should be absolutely avoided; alcoholic beverages and stimulating foods or spices interdicted.

Our attention should be directed toward the general as well as the local treatment. To obviate the possibility of complications, thereby checking the spread of the microörganisms beyond the urethral area besides keeping the urine in a more aseptic condition, the patient should be at once given sandalwood oil internally. The therapeutic value of this drug is so well-known as to require no special comment beyond saying that our success with it depends altogether upon the variety and quality of the oil used. From our own clinical observation in a vast number of cases treated at the dispensary and in private practice, santyl, which is the neutral salicylic ester of santalol, deserves special mention. Here may be noted a few of the many cases:

Case 1.—E. R., age 30; tailor. Discharge three days' duration; extreme pain in micturition; profuse purulent discharge. After two days' administration of santyl m xv. q. four hours, pain began to subside and disappeared entirely at the end of the week; the discharge also became much thinner and scantier; at the end of the second week experienced no abnormal sensation in the urethra and bore the injection of protargol perfectly well. At no time was there any gastric disturbances.

Case 2.—A. L., age 19; electrician; presented himself on the first day of the onset of disease; glans very much edematous marked ardor and chordeé; santyl at once given in doses m. xxi. q. four hours; all acute symptoms began to subside on the second day—at the end of the week began local treatment. At this time patient felt very comfortable; the discharge diminished from day to day and in the beginning of the third week ceased entirely. There was total absence of gonococci in the secretion. Expressed by prostatic massage; internal medication and local treatment still continued, however, for three weeks longer. For two weeks more there was no return of any of the symptoms although the patient was kept for further observation.

Case 3.—J. C., age 29; tailor; discharge one week standing; complained of painful and frequent micturition and very frequent erections which annoyed him very much; also of pain in the pit of stomach saying that a friend had advised him to take the Lafayette mixture. There was no marked local inflammation, so he was given at once santyl in capsules aa m. vii., 3 q. four hours. Two days later when he returned all uncomfortable feeling about the penis disappeared entirely; he slept better, and altogether the drug was well borne by the stomach. He was given anterior irrigation with protargol 1½ per cent. every other day and at the end of a week his urine cleared, and at the end of two weeks more discharge entirely ceased. He thought himself entirely cured and we lost all trace of him for four months.

Case 4.—A. J., chauffeur, age 28; first attack two years ago; present trouble three days' standing; complained of intense burning, perineal pains and constant discharge; santyl given in m. xxx. q. four hours. Borne very well by the stomach; all pains disappeared entirely at the end of three days. Local treatment with protargol begun; the urine gradually cleared; discharge ceased at the end of ten days. Treatment continued for four weeks more; then told to return in ten days; he indulged in beer quite freely; there was no return of the symptoms.

Case 5.—J. S., age 40; laborer; discharge five days' duration; marked ardor; profuse discharge, at times bloody. Santyl m. xxi. q. four hours in capsules, symptoms gradually began to subside on the second day and at the end of the fourth day discharge became mucoid and quite scanty; was able to commence local treatment. Had no gastric disturbance at any time and in four weeks made an uneventful recovery. Was not seen for two months when he returned with a chancroid on the penis. The urine seen at this time was perfectly clear; there was no discharge.

Case 6.—M. R., age 23; tailor; discharge two days' standing. Intense scalding; almost constant erections; santyl m xv. q. four hours given. In two days felt entirely relieved; no gastric disturbance, so dose was increased to m xxi. q. four hours; anterior irrigation with protargol ½ per cent. every day begun; urine then began to clear from day to day. On the twelfth day of the disease there was no more discharge, the urine was clear. After six weeks' treatment there was no recurrence of symptoms although patient was kept under observation.

Case 7.—A. L., age 21; clerk; first attack; discharge four days' standing, marked ardor and profuse discharge; santyl in m xv. doses q. four hours. On the following day symptoms markedly relieved, on the third day entirely gone. Anterior irrigation with protargol ½ per cent. b. i. d. In two weeks discharge entirely ceased and urine cleared up. He then left for the country and took along sufficient number of capsules to last for two weeks. During this time he received no local treatment. Two months later he returned with no indication of the disease.

Case 8.—B. B., plumber. age 28; single; first attack; marked swelling of glans and pain in the groins; profuse thick purulent discharge; all these symptoms quickly subsided, there being no gastric disturbance. On the seventh day after having received daily irrigation q. protargol ½ per cent. and santyl in xxi, m. doses q. four hours, discharge almost entirely ceased, appearing only in the morning. On the tenth day had no discharge, although the urine persisted slightly cloudy for one week during which time I increased santyl to four capsules aa m vii., q. four hours. Urine then gradually cleared, and during the following three weeks the urine was absolutely clear.

Case 9.—J. C., age 18; student; first attack; purulent discharge; three days' standing; marked ardor and frequent micturition; santyl xiv. m. q. four hours. On the third morning began to show marked improvement, in that burning entirely ceased; at the end of the second week after daily anterior irrigation of protargol ½ per cent. discharge entirely disappeared and for over three weeks did not recur.

As will be noted from the foregoing cases there was a remarkable total absence of complications; other cases are too numerous to mention, and almost without exception we found santyl most valuable and efficacious in that it never produced any untoward effects upon the stomach, kidneys or other organs, and it is also worth mentioning that it imparted no odor to the breath or urine as is common with the sandalwood oil usually employed. That this neutral salicylic ester of santalol exerts a far better influence on the disease than copaiba or any oleoresinous balsam is beyond question. It may also be added that it is advantageous to continue the internal medicament for some weeks after the total disappearance of the discharge.

As for local treatment we must bear in mind the importance of destroying the gonococci, while inflicting as little injury to the tissues involved as possible, at the same time healing and assisting the structures in combating the further progress of the disease. To accomplish this requires more or less skilful technic and protargol is about the best therapeutic measure at our command. Although silver nitrate, the old and tried remedy, is quite efficacious and is perhaps preferred by a great many, still protargol stands first, in that it possesses far more powerful germicidal properties, its more profound penetration into the tortuous crypt and deep recesses of the tissues, and its freedom from irritating and injurious effects upon the mucous membrane.

Something must also be said regarding the frequent employment of astringents. To begin with an astringent is to invite the multiplication of the gonococci, and an invasion of the deep-seated tissues because of their protection in the folds and ducts of the mucous membrane, where astringents which are seldom germicides cannot reach them. Just as soon as the intensity of the acute inflammation has subsided, and this can only be accomplished by careful diet, rest and the judicious employment of the santyl, then should anterior irrigation of protargol in the strength of ½ to 1 per cent. be begun twice a day, or where impracticable once a day. Hand injections by the patient may be good but often these prove injurious and should not be wherever possible. The patient should be instructed to urinate into two clean glasses prior to injection and the appearance of the specimens noted from day to day before each treatment is administered. Usually after a few days careful local and general treatment the ardor gradually diminishes and quickly disappears; the discharge becomes mucoid in character and scanty, and microscopical examination often reveals a rapid diminution in the number of the gonococci until finally these cannot be revealed. Treatment should, however, be continued, patient kept for observation and repeated examination of the urine and prostatic secretion if any, be made long after apparent cure at intervals of weeks.

1184 Madison Avenue, New York City.

